# Seizure reduction is a prognostic marker in low-grade glioma patients treated with temozolomide

**DOI:** 10.1007/s11060-015-1975-y

**Published:** 2015-11-07

**Authors:** Johan A. F. Koekkoek, Linda Dirven, Jan J. Heimans, Tjeerd J. Postma, Maaike J. Vos, Jaap C. Reijneveld, Martin J. B. Taphoorn

**Affiliations:** Department of Neurology, VU University Medical Center, Amsterdam, The Netherlands; Department of Neurology, Medical Center Haaglanden, The Hague, The Netherlands; Department of Neurology, Leiden University Medical Center, PO Box 9600, 2300 RC Leiden, The Netherlands

**Keywords:** Seizures, Chemotherapy, Temozolomide, Primary brain tumor, Glioma

## Abstract

We aimed to analyze the value of seizure reduction and radiological response as prognostic markers of survival in patients with low-grade glioma (LGG) treated with temozolomide (TMZ) chemotherapy. We retrospectively reviewed adult patients with a progressive LGG and uncontrolled epilepsy in two hospitals (VUmc Amsterdam; MCH The Hague), who received chemotherapy with TMZ between 2002 and 2014. End points were a ≥50 % seizure reduction and MRI response 6, 12 and 18 months (mo) after the start of TMZ, and their relation with progression-free survival (PFS) and overall survival (OS). We identified 53 patients who met the inclusion criteria. Seizure reduction was an independent prognostic factor for both PFS (HR 0.38; 95 % CI 0.19–0.73; p = 0.004) and OS (HR 0.39; 95 % CI 0.18–0.85; p = 0.018) after 6mo, adjusting for age and histopathological diagnosis, as well as after 12 and 18mo. Patients with an objective radiological response showed a better OS (median 87.5mo; 95 % CI 62.0–112.9) than patients without a response (median 34.4mo; 95 % CI 26.1–42.6; p = 0.046) after 12mo. However, after 6 and 18mo OS was similar in patients with and without a response on MRI. Seizure reduction is an early and consistent prognostic marker for survival after treatment with TMZ, that seems to precede the radiological response. Therefore, seizure reduction may serve as a surrogate marker for tumor response.

**Electronic supplementary material:**

The online version of this article (doi:10.1007/s11060-015-1975-y) contains supplementary material, which is available to authorized users.

## Introduction

Diffuse low-grade gliomas (LGGs) are slowly growing, diffuse infiltrative brain tumors, including astrocytomas, mixed oligoastrocytomas and oligodendrogliomas, that mainly affect young adults [1, 2]. Median survival generally ranges between 2 and 20 years, depending on histopathological subtype, age, tumor size and performance status [3–5].

Assessing response to antitumor treatment is a major challenge in LGG patients. Diffuse LGGs are predominantly non-contrast enhancing diffusely infiltrative tumors, hampering radiographic assessment. Complete radiological responses are rare, and symptomatic improvement is not always accompanied by a radiological response [6–8]. Therefore, the use of objective radiological response as an outcome measure has serious drawbacks, and there is a strong need for complementary measures to determine outcome and to define the benefit of antitumor treatment [8].

Seizures often are the sole clinical symptom of a diffuse LGG, occurring in 70–90 % of patients during the course of the disease [9–11]. Apart from treatment with antiepileptic drugs (AEDs), antitumor treatment itself may contribute to a reduction in seizure frequency. Seizure reduction has been reported both after focal fractionized irradiation and after chemotherapy with either procarbazine, lomustine and vincristine (PCV) [12] or temozolomide (TMZ) [13–16]. Furthermore, several studies showed that a reduction in seizure frequency may occur in the absence of a radiological response on MRI after treatment [13, 15, 17–19]. We previously showed that a seizure reduction 6 months (mo) after the start of TMZ is associated with a longer survival in LGG patients [20]. Possibly, seizure reduction may serve as an additional outcome measure for tumor response after antitumor treatment. However, its precise value in comparison with radiological response is still unknown.

In the current study, we analyzed the value of seizure reduction and radiological response as prognostic markers of survival in patients with progressive LGG and uncontrolled epilepsy that were treated with TMZ.

## Patients and methods

### Study population

We studied adult (≥18 years) patients with a grade II glioma according to the World Health Organization (WHO) criteria, treated at the Medical Center Haaglanden, The Hague, or VU University Medical Center, Amsterdam, between January 2002 and January 2014. Using institutional databases, we selected patients who met the following inclusion criteria: (1) histopathological diagnosis of a supratentorial WHO grade II astrocytoma, oligoastrocytoma or oligodendroglioma; (2) radiological and/or clinical signs of tumor progression; (3) treatment with TMZ for at least 6 cycles, or until renewed tumor progression or unacceptable toxicity emerged; (4) presence of uncontrolled epilepsy, defined as at least 1 seizure 3mo before the start of TMZ, despite use of AEDs and not attributable to subtherapeutic AED serum levels; (5) no other antitumor therapy during TMZ treatment; (6) availability of follow-up data on seizure outcome after 6, 12 or 18mo and on survival. We excluded patients with an increased AED dose or switch in AED regimen during TMZ treatment.

### Patients

We collected additional data from the hospital medical charts on date and type of first symptoms at diagnosis, antitumor treatment before and after TMZ, seizure semiology and frequency and steroid use. We classified seizures as partial (simple or complex), generalized, or a combination of both. We divided seizure frequency into two categories, based on the median score: ≥1 seizure/week or <1/week. Regarding the different types of surgery, we made a distinction between gross-total resection, partial resection and a biopsy. Gross-total resection was defined as the absence of residual tumor during surgery and on postoperative T2-weighted MRI. In case of any residual tumor, resections were classified as partial. The local ethics committee approved the study protocol.

### Study end points

We evaluated the presence of a ≥50 % seizure reduction 6, 12 or 18mo after the start of TMZ treatment. A ≥50 % seizure reduction was defined as a reduction of ≥50 % in the number of seizures per unit of time compared to baseline. All other seizure outcomes (a <50 % reduction in the number of seizures, a similar or an increased seizure frequency) were considered a <50 % seizure reduction. Seizure reduction was based on seizure frequencies reported by the patient, without electroencephalographic confirmation.

We analyzed progression-free survival (PFS), defined as duration of survival from the start of TMZ to clinical or radiological progression, and overall survival (OS), defined as duration of survival from the start of TMZ until death, using the response assessment in neuro-oncology (RANO) criteria [8].

To analyze the radiological response, two investigators (JAFK and LD) independently measured tumor response on MRI. In line with the RANO criteria for LGG, we defined complete response (CR) as a complete disappearance of the lesion, partial response (PR) as a ≥50 % decrease and minor response (MR) as a 25–50 % decrease of the area of the lesion. Patients with a CR, PR or MR (i.e. an objective response) had to be stable or improved clinically without an increased corticosteroid dose. Progressive disease (PD) was defined as a ≥25 % increase in the area of the lesion, or the development of new lesions, or increased or new enhancement. Stable disease (SD) was defined as any status other than CR, PR, MR or PD. To measure the differences in the area of the lesion, we used the change in the product of the perpendicular T2 tumor diameters between MR imaging at 6, 12 and 18mo, and the imaging at baseline. In case of disagreement, which we defined as a >10 % difference in the product of the perpendicular diameters, the two investigators performed a consensus measurement through discussion.

### Statistical analysis

We performed statistical analysis using SPSS V.21.0 software. We defined the study population using descriptive statistics. Differences between patients with and without a seizure reduction were tested with the χ^2^ or Fisher’s exact test for categorical data and the Student t test for continuous data. We explored both seizure outcome and radiological response in relation to survival (PFS and OS), using Kaplan–Meier estimates and log-rank tests. Multivariable Cox modelling was applied to determine the association between seizure reduction and survival, taking into account two well-known prognostic factors (age and histopathological diagnosis). We censored patients for survival analysis at the end of the study (July 1, 2014) who showed no evidence of renewed tumor progression after treatment with TMZ or who were still alive.

## Results

### Patient characteristics

We identified 53 patients who met all the in- and exclusion criteria. Thirteen patients were excluded due to an increase in AED dose or a switch in AED type. Baseline characteristics are outlined in Table 1. The cohort consisted of 32 patients (60.4 %) with astrocytoma, 7 (13.2 %) with oligoastrocytoma and 14 with oligodendroglioma (26.4 %).

**Table 1:** Baseline characteristics

	All patients (n = 53) n (%)
Gender	
Male	28 (52.8)
Female	25 (47.2)
Mean age at start TMZ, y (SD)	47.3 (12.0)
Tumor type	
Astrocytoma	32 (60.4)
Oligoastrocytoma	7 (13.2)
Oligodendroglioma	14 (26.4)
Tumor location	
Frontal	29 (54.7)
Temporal	11 (20.8)
Parietal	7 (13.2)
Occipital	1 (1.9)
Basal ganglia/midline	5 (9.4)
Median time from first symptoms to start of TMZ, years (IQR) (n = 52)	5.8 (5.7)
Other focal symptoms at diagnosis (n = 51)	14 (27.5)
Extent of surgery	
Gross-total resection	5 (9.4)
Partial resection	25 (47.2)
Biopsy	23 (43.4)
Radiotherapy	38 (71.7)
Seizure classification	
Partial simple	23 (43.4)
Partial complex	7 (13.2)
Secondary generalized	15 (28.3)
Both partial and generalized	8 (15.1)
Seizure frequency	
≥1/week	22 (41.5)
<1/week	31 (58.5)
AED polytherapy	34 (64.2)
Steroid use	27 (50.9)

Twenty-three patients (43.4 %) had simple partial seizures, 7 (13.2 %) complex-partial, 14 (28.3 %) generalized and 8 (15.1 %) had a combination of both partial and generalized seizures. Twenty-two patients (41.5 %) reported more than one seizure a week; 31 patients (58.5 %) had less than one seizure a week. All 53 patients received AED treatment when TMZ treatment was initiated and 34 patients (64.2 %) were on AED polytherapy at that time.

All patients underwent surgery prior to TMZ treatment: in 5 patients (9.4 %) a gross-total resection had been performed, in 25 (47.2 %) a partial resection and in 23 patients (43.4 %) a biopsy only. Thirty-eight patients (71.7 %) had received focal fractionated irradiation before the start of TMZ treatment. The median time between the end of radiotherapy and the start of TMZ was 3.9 years (range 0.8–13.9 years). Twelve patients (22.6 %) underwent a second resection due to tumor progression. Median time from first symptoms to the start of TMZ was 5.8 years (Inter Quartile Range (IQR) 5.7).

Median follow-up time from the start of TMZ was 32.1mo (IQR 34.3). At last follow-up, 47/53 patients (88.7 %) had developed tumor recurrence with a median PFS of 20.0mo (95 % CI:13.8–26.2), and 32/53 patients (60.4 %) had died, showing a median OS of 39.1mo (95 % CI:27.2–50.9). Within 6mo after initiation of TMZ, 2/53 patients had developed tumor recurrence, of which 1 patient had died. Within 12mo, a total of 12/53 patients had developed tumor recurrence, of which 5 patients had died. Within 18mo, a total of 16/53 patients had developed tumor recurrence, of which 8 patients had died. Three patients in whom seizure outcome was missing at 12 and 18mo were excluded from further analysis. As a consequence, at 6, 12 and 18mo, 51, 38 and 34 patients were available for the analysis of seizure outcome and survival, respectively (Supplementary figure).

### Seizure outcome

Six, 12 and 18mo after initiation of TMZ treatment, 25/51 (49.0 %), 24/38 (63.2 %) and 21/34 (61.8 %) patients showed a ≥ 50 % seizure reduction, respectively. Of the 34 patients without tumor progression within 18mo, 17 (50.0 %) patients had a persistent ≥50 % seizure reduction at 6, 12 as well as 18mo (Supplementary Fig. 1).

Various demographic and clinical variables were compared between patients with and without seizure reduction, and are outlined in Table 2. There were no statistically significant differences between patients with and without a ≥50 % seizure reduction at 6, 12 and 18mo after the start of TMZ. AEDs were withdrawn within 18mo after the start of TMZ in 4 patients with a ≥50 % seizure reduction, and in 2 other patients AED dose was reduced. In patients without seizure reduction, AED dose was reduced in 1 patient within 18mo.

**Table 2:** Differences in demographic and clinical characteristics between patients with and without seizure reduction at 6, 12 and 18 months after the start of temozolomide

	Seizure reduction at 6mo (n = 51)			Seizure reduction at 12mo (n = 38)			Seizure reduction at 18mo (n = 34)		
	<50 %	≥50 %	p value	<50 %	≥50 %	p value	<50 %	≥50 %	p value
All patients	26 (50.9)	25 (49.1)		14 (36.8)	24 (63.2)		13 (38.2)	21 (61.8)	
Mean age, y (SD)	47.5 (12.6)	46.0 (11.5)	0.65	50.7 (10.8)	45.5 (11.6)	0.18	49.2 (13.4)	45.5 (11.0)	0.39
Tumor type			0.82			0.27			0.84
Astrocytoma	15 (57.7)	15 (60.0)		8 (57.1)	12 (50.0)		7 (53.8)	11 (52.4)	
Oligoastrocytoma	3 (11.5)	4 (16.0)		0	4 (16.7)		1 (7.7)	3 (14.3)	
Oligodendroglioma	8 (30.8)	6 (24.0)		6 (42.9)	8 (33.3)		5 (38.5)	7 (33.3)	
Other focal symptoms at diagnosis (n = 49)	4 (16.0)	9 (37.5)	0.088	1 (7.7)	6 (26.1)	0.38	1 (3.5)	5 (25.0)	0.37
Extent of surgery			0.33			0.72			0.28
Resection	17 (65.4)	13 (52.0)		9 (64.3)	14 (58.3)		10 (76.9)	11 (52.4)	
Biopsy	9 (34.6)	12 (48.0)		5 (35.7)	10 (41.7)		3 (23.1)	10 (47.6)	
Radiotherapy	20 (76.9)	16 (64.0)	0.31	12 (85.7)	14 (58.3)	0.080	11 (84.6)	12 (57.1)	0.096
Seizure classification			0.12			0.94			0.64
Partial	12 (46.2)	17 (68.0)		8 (57.1)	14 (58.3)		7 (53.8)	13 (61.9)	
Generalized or both partial and generalized	14 (53.8)	8 (32.0)		6 (42.9)	10 (41.7)		6 (46.2)	8 (38.1)	
Seizure frequency			0.33			0.74			0.17
>1/week	17 (65.4)	13 (52.0)		8 (57.1)	15 (62.5)		6 (46.2)	15 (71.4)	
<1/week	9 (34.6)	12 (48.0)		6 (42.9)	9 (37.5)		7 (53.8)	6 (28.6)	
AED polytherapy	18 (69.2)	15 (60.0)	0.49	10 (71.4)	16 (66.7)	1.00	10 (76.9)	14 (66.7)	0.70
Steroid use	13 (50.0)	12 (48.0)	0.89	6 (42.9)	9 (37.5)	0.74	7 (53.8)	6 (28.6)	0.17

### Seizure reduction and survival

**Fig. 1:**
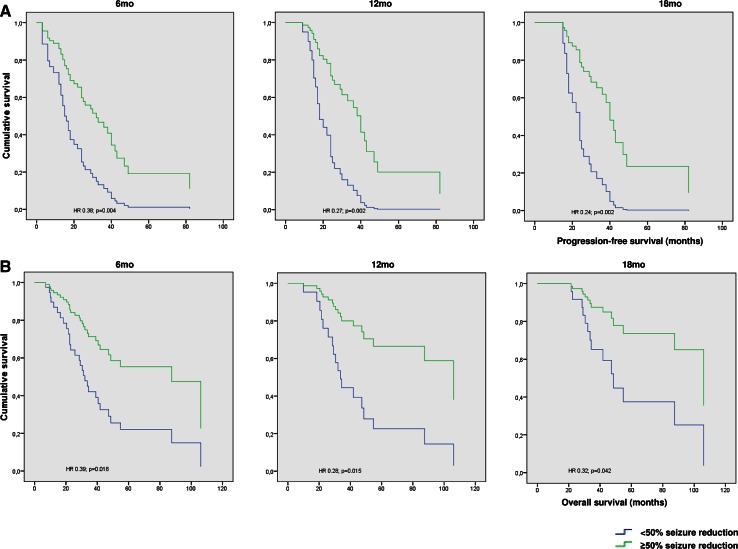
Seizure reduction 6, 12 and 18mo after the start of TMZ in relation to progression-free survival (**a**) and overall survival (**b**)

Patients with a ≥50 % seizure reduction at 6mo showed a significantly longer median PFS of 26.0mo (95 % CI 15.7–36.3) compared to a median PFS of 14.0mo (95 % CI 8.0–20.0) in patients without seizure reduction (HR 0.38; 95 % CI 0.19–0.73; p = 0.004), after adjustment for age and histopathological diagnosis (Fig. 1; supplementary Table 1). At 12mo after the start of TMZ, a ≥50 % seizure reduction also was an independent prognostic factor for PFS (HR 0.27; 95 %CI 0.12–0.62; p = 0.002), as well as at 18mo (HR 0.24; 95 % CI 0.10–0.59; p = 0.002).

Patients with a ≥50 % seizure reduction at 6mo showed a significantly longer median OS of 87.5mo (95 % CI 35.8–139.1) compared to a median OS of 30.5mo (95 % CI 20.3–40.7) in patients without seizure reduction (HR 0.39; 95 % CI 0.18–0.85; p = 0.018), adjusting for age and histopathological diagnosis (Fig. 1; supplementary Table 1). At 12mo after the start of TMZ, a ≥50 % seizure reduction also was an independent prognostic factor for OS (HR 0.28; 95 % CI 0.097–0.77; p = 0.015), as well as at 18mo (HR 0.31; 95 % CI 0.10–0.96; p = 0.042).

We evaluated the use of antitumor treatment after administration of TMZ, as it might have had an effect on OS as well. In 29/47 patients (61.7 %) who developed tumor recurrence after TMZ treatment, new antitumor treatment was initiated. A partial resection was performed in 10/29 cases, of which 7 revealed a malignant transformation to a WHO grade III tumor (3 cases) or glioblastoma (4 other cases). Re-challenge with TMZ was started in 12 cases, radiotherapy in 8 cases, combined radio-chemotherapy with TMZ in 3, a combination of chemotherapy with PCV in 5, and lomustine monotherapy in 1 case. Twelve patients received a combination of 2 or more treatments until the last follow-up. At 6, 12 and 18mo, there was no statistically significant difference between patients with and without a ≥50 % seizure reduction with regard to the initiation of new antitumor treatment after TMZ.

### Radiological response and its association with seizure reduction and survival

At 6mo, 11/50 patients (22.0 %) showed an objective response to treatment, compared to 23/37 patients (62.2 %) at 12mo, and 21/33 patients (63.6 %) at 18mo. We found no CRs at 6, 12 and 18mo (supplementary Table 2).

The radiological response in relation to seizure reduction is outlined in Table 3. After 6mo, 7/24 patients (29.2 %) with a ≥50 % seizure reduction had an objective response on MRI, compared to 4/26 patients (15.4 %) without a seizure reduction (p = 0.24). After 12mo 18/23 patients (78.3 %) with a ≥50 % seizure reduction had an objective response on MRI, compared to 5/14 patients (35.7 %) without a seizure reduction (p = 0.010). After 18mo 13/21 patients (61.9 %) with a ≥50 % seizure reduction had an objective response, compared to 7/12 patients (58.3 %) without a seizure reduction (p = 0.84). Of the 24 patients with a ≥50 % seizure reduction at 6mo, 15 patients (62.5 %) showed an objective response on MRI at 12mo, compared to 9/26 patients (34.6 %) without a seizure reduction at 6mo (p = 0.049). Fourteen of 24 patients (58.3 %) with a ≥50 % seizure reduction at 6mo showed an objective response on MRI at 18mo, compared to 7/26 patients (26.9 %) without a seizure reduction at 6mo (p = 0.025).

**Table 3:** Radiological response in relation to seizure reduction

	6 months (n = 50)		12 months (n = 37)		18 months (n = 33)	
Seizure reduction	No response	Response	No response	Response	No response	Response
<50 % seizure reduction	22	4	9	5	5	8
≥50 % seizure reduction	17	7	5	18	7	13

**Fig. 2:**
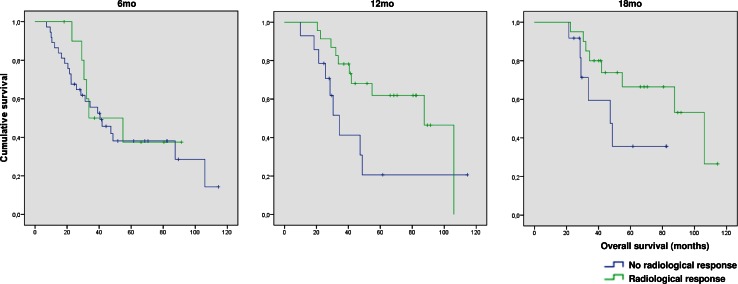
Radiological response 6, 12, and 18mo after the start of TMZ in relation to overall survival

We analyzed the prognostic value of an objective response on MRI for OS (Fig. 2). At 6mo, OS was similar in patients with (median 33.6mo; 95 % CI 2.4–64.9) and without (median 40.5mo; 95 % CI 24.8–56.3; p = 0.640) an objective response on MRI. At 12mo, patients with an objective response showed a better OS (median 87.5mo; 95 % CI 62.0–112.9) than patients without an objective response (median 34.4mo; 95 % CI 26.1–42.6; p = 0.046). At 18mo, we found no statistical difference in OS between patients with (median 106.1mo; 95 % CI 55.1–157.1) and without an objective response (median 47.3; 95 % CI 26.7–67.9; p = 0.096).

## Discussion

TMZ is currently the preferred chemotherapy in patients with a progressive LGG, as it is well-tolerated and relatively easy to administrate [21]. Response to TMZ treatment based on radiological assessment is difficult in LGG, as on MRI a minor response is often the best achievable response category and measuring tumor size can be challenging [7].

In the present study, we compared seizure reduction with the response on MRI as prognostic markers for survival in patients with a progressive LGG treated with TMZ. Seizure reduction appeared to be a consistent marker for a survival benefit in patients with progressive LGG up to 18mo after the start of TMZ. More importantly, after 6mo, patient’s seizure status reflected both PFS and OS, in contrast to radiological response. Furthermore, a ≥50 % seizure reduction at 6mo was associated with the occurrence of an objective MRI response at both 12mo and 18mo. No less than 8/17 patients with a seizure reduction but no MRI response at 6mo, showed a radiological response 6mo later. So, seizure reduction not only seems to be a favorable prognostic marker for survival at different time points from the start of TMZ, but also seems to precede the radiological response. In other words, a ≥50 % seizure reduction appears to be a relatively early sign that the tumor is responding to treatment.

Our data suggest that absence of a radiological response should not prevent physicians from continuing TMZ treatment. Previous studies in LGG support our findings that radiological responses to chemotherapy are often slow and delayed [21]. In a series of 149 patients with LGG treated with up-front TMZ, 77 (53 %) showed an objective response on MRI with a median time to maximum response of 12mo (range 3–30mo) [16]. In a study of 21 patients treated with PCV an ongoing decrease in tumor size was observed in 20 cases, which sometimes lasted for years after discontinuation of treatment [22]. So the observed radiological response rate largely depends on the timing of the assessment. Therefore, response on MRI could underestimate the benefit of the treatment, particularly in the early phase after initiation of TMZ [7]. In addition, the first 25 % decrease in the area of the lesion does not qualify for a minor response according to the RANO criteria [8, 23]. As a consequence, there will be some patients with slowly decreasing tumor size who are regarded as non-responders during the first months of antitumor treatment.

Altogether, the observed radiological response is not always related to survival in LGG patients. This is supported by our data in which the objective MRI response was not associated with a better OS after 6 and 18mo. Although the use of more advanced imaging techniques, such as MRI measuring tumor volume or growth rate, may also help to overcome current flaws in assessing radiological response to treatment, a marker like seizure reduction has the advantage of being easily recordable and applicable in clinical practice without much effort.

Apart from being a prognostic marker for successful antitumor treatment, seizure reduction in itself means a clinical benefit for the patient with an LGG, as it may contribute to an improved quality of life and better neurocognitive functioning [24–26]. A reduction in seizure frequency after TMZ treatment has previously been described in 44–62 % of patients [13, 14, 16, 17, 20, 27]. Seizure freedom after radiotherapy has been found in 25–75 % of patients [15, 28, 29]. The precise mechanism of action through which antitumor treatment affects seizure status is still unknown. A reduction in the intrinsic epileptogenicity of the tumor, by affecting the glutamate metabolism or induction of changes in the peritumoral microenvironment could play a role [30, 31]. Despite the fact that after 6mo an objective radiological response was detected in only a minority of patients with a seizure reduction, seizure reduction could result from a decrease in tumor size as well, albeit a small one. After all, seizure reduction after 6mo was strongly related to the occurrence of a tumor response on MRI later on, after 12 and 18mo.

Our current study has several limitations. First, reports on seizure frequency are prone to recall and reporting bias, which is inherent to the retrospective nature of the study. Second, because of our relatively small patient population we were unable to perform separate analyses for each type of radiological response. For the same reason, we were neither able to perform analyses after more than 18mo of follow-up, which could have further strengthened our findings. Third, we included patients with a progressive LGG who had previously been treated with various types of antitumor treatment, contributing to a relatively heterogeneous patient population. In addition, patients with unresectable tumors were overrepresented in our population, given the 43.4 % of patients who had undergone a biopsy before TMZ treatment, which is higher than the general LGG population [32]. Apart from that, patient characteristics were representative of LGG patients [33, 34], and were equally distributed among patients with and without a seizure reduction. Furthermore, we selected only patients without concurrent antitumor treatment and without changes in the AED regimen that could influence seizure outcome, eliminating major factors that could have affected seizure outcome.

In conclusion, this is the first study that identifies seizure reduction in LGG patients treated with TMZ both as a prognostic marker for survival, and as a marker which precedes MRI tumor response. We believe that seizure reduction is suitable as a complementary outcome measure along with radiological response in LGG patients with uncontrolled epilepsy. Future prospective studies should aim at a validation of patient’s seizure status as a surrogate marker for tumor response during antitumor treatment.

## Electronic supplementary material

Supplementary material 1 (DOC 99 kb)

Supplementary material 2 (DOC 49 kb)

Supplementary material 3 (DOC 39 kb)
