# Supplementary material for: Seizure reduction is a prognostic marker in low-grade glioma patients treated with temozolomide
Source: J Neurooncol. 2015 Nov 7;126:347–54. doi: 10.1007/s11060-015-1975-y (PMC4718947; doi:10.1007/s11060-015-1975-y)
Supplement: Supplementary file 1 — Supplementary material 1 (DOC 99 kb) [file 11060_2015_1975_MOESM1_ESM.doc]

Patients available for analysis

**53**

**6 months**

<50% seizure reduction

**26**

≥50% seizure reduction

**25**

Progression 2

(1 died)

**12 months**

**18 months**

Progression 10

(4 died)

<50% seizure reduction

**14**

13

21

3

1

20

4

1

9

<50% seizure reduction

**13**

≥50% seizure reduction

**21**

≥50% seizure reduction

**24**

Progression 4

(3 died)

Missing 3
