# Supplementary material for: Seizure reduction is a prognostic marker in low-grade glioma patients treated with temozolomide
Source: J Neurooncol. 2015 Nov 7;126:347–54. doi: 10.1007/s11060-015-1975-y (PMC4718947; doi:10.1007/s11060-015-1975-y)
Supplement: Supplementary file 2 — Supplementary material 2 (DOC 49 kb) [file 11060_2015_1975_MOESM2_ESM.doc]

**Supplementary table 1: multivariable Cox modelling of survival**

|  | **Progression-free survival** | | | | | | | | |
| --- | --- | --- | --- | --- | --- | --- | --- | --- | --- |
| **Prognostic factor** | **6mo after start TMZ (n=51)** | | | **12mo after start TMZ (n=38)** | | | **18mo after start TMZ (n=34)** | | |
|  | **HR** | **95% CI** | **p-value** | **HR** | **95% CI** | **p-value** | **HR** | **95% CI** | **p-value** |
| Seizure reduction ≥50% | 0.38 | 0.19-0.73 | ***0.004*** | 0.27 | 0.12-0.62 | ***0.002*** | 0.24 | 0.10-0.59 | ***0.002*** |
| Oligodendroglioma vs astrocytoma | 0.39 | 0.18-0.83 | ***0.015*** | 0.62 | 0.28-1.40 | 0.25 | 0.43 | 0.17-1.05 | 0.065 |
| Age at diagnosis | 1.01 | 0.98-1.04 | 0.46 | 1.02 | 0.98-1.05 | 0.39 | 1.01 | 0.98-1.05 | 0.55 |
|  |  |  |  |  |  |  |  |  |  |
|  | **Overall survival** | | | | | | | | |
| **Prognostic factor** | **6mo after start TMZ (n=51)** | | | **12mo after start TMZ (n=38)** | | | **18mo after start TMZ (n=34)** | | |
|  | **HR** | **95% CI** | **p-value** | **HR** | **95% CI** | **p-value** | **HR** | **95% CI** | **p-value** |
| Seizure reduction ≥50% | 0.39 | 0.18-0.85 | ***0.018*** | 0.28 | 0.097-0.77 | ***0.015*** | 0.32 | 0.10-0.96 | ***0.042*** |
| Oligodendroglioma vs astrocytoma | 0.24 | 0.080-0.72 | ***0.011*** | 0.38 | 0.12-1.23 | 0.11 | 0.20 | 0.040-0.98 | ***0.046*** |
| Age at diagnosis | 1.02 | 0.98-1.05 | 0.33 | 1.02 | 0.97-1.06 | 0.45 | 1.02 | 0.97-1.07 | 0.42 |
