# Supplementary material for: Seizure reduction is a prognostic marker in low-grade glioma patients treated with temozolomide
Source: J Neurooncol. 2015 Nov 7;126:347–54. doi: 10.1007/s11060-015-1975-y (PMC4718947; doi:10.1007/s11060-015-1975-y)
Supplement: Supplementary file 3 — Supplementary material 3 (DOC 39 kb) [file 11060_2015_1975_MOESM3_ESM.doc]

**Supplementary table 2: radiographic findings on MRI**

| **Response on MRI** | **6 months**  (n=50) | **12 months**  (n=37) | **18 months**  (n=33) |
| --- | --- | --- | --- |
| **Progressive disease** | 12 (24.0) | 3 (8.1) | 8 (24.2) |
| **Stable disease** | 27 (54.0) | 11 (29.7) | 4 (12.1) |
| **Minor response** | 8 (16.0) | 11 (29.7) | 11 (33.3) |
| **Partial response** | 3 (6.0) | 12 (32.4) | 10 (30.3) |
